# Impact of Sarcopenia on Survival in Patients With Early-Stage Lung Cancer Treated With Stereotactic Body Radiation Therapy

**DOI:** 10.7759/cureus.10712

**Published:** 2020-09-29

**Authors:** James M Taylor, Andrew Song, Allison R David, Victor E Chen, Bo Lu, Maria Werner-Wasik

**Affiliations:** 1 Radiation Oncology, Sidney Kimmel Cancer Center at Thomas Jefferson University, Philadelphia, USA; 2 Radiation Oncology, Thomas Jefferson University Hospital, Philadelphia, USA; 3 Radiation Oncology, Sidney Kimmel Medical College, Philadelphia, USA; 4 Internal Medicine, Boston Medical Center, Boston, USA

**Keywords:** stereotactic ablative radiation, sarcopenia, non-small cell lung cancer

## Abstract

Background

Sarcopenia has been associated with poor survival among cancer patients. Normalized total psoas area (NTPA) has been used as a surrogate for defining sarcopenia. Few data exist characterizing the impact of sarcopenia and other metrics of fitness on clinical outcomes in patients with early-stage non-small cell lung cancer (NSCLC) treated non-invasively with stereotactic body radiotherapy (SBRT).

Methods

To assess the association between sarcopenia and clinical outcomes, we conducted a retrospective analysis of consecutive patients treated with SBRT from 2013 to 2019 . Overall survival (OS), local failure free survival (LFS), distant failure free survival (DFS), NTPA, body mass index (BMI), and Charlson comorbidity index (CCI) were included for analysis. NTPA was calculated by measuring the psoas volume at the L3 vertebra and normalizing for patient height and gender. Survival functions were evaluated using the Kaplan-Meier method. Log-rank test and Cox-proportional hazards were performed for categorical and continuous variables, respectively. Significance was set as p < 0.05.

Results

A total of 91 patients met the criteria. The median age was seven years and Karnofsky Performance Status score (KPS) was 80 (range: 60-100). Approximately 79% of patients had T1 tumors. Median radiation dose and number of fractions were 60 Gy (range: 45-60) and 5 fractions (range: 3-5). Median NTPA was 531.16 mm^2^/m^2 ^(range: 90.4-1356.2). After normalization (sarcopenia: <385 mm^2^/m^2^, female; <585 mm^2^/m^2, ^male), 39 patients (42.8%) had sarcopenia. NTPA had no association with OS (p = 0.7), LFS (p = 0.9), or DFS (p = 0.5). Increasing BMI was associated with improved OS (HR 0.90, 95% CI 0.83-0.98). With a median follow-up of 23.4 months, median OS was 60, 60, and 45.9 months (p = 0.37) in all patients, non-sarcopenic patients, and sarcopenic patients, respectively.

Conclusion

Sarcopenia was not associated with OS, LFS, or DFS. Increasing BMI is associated with improved OS. Future, prospective work is needed to define the impact of sarcopenia and other fitness metrics on clinical outcomes among NSCLC patients treated non-invasively with SBRT.

## Introduction

Lung cancer is the second most common malignancy in the United States, second only to prostate and breast cancer in men and women, respectively [1]. However, lung cancer is the leading cause of cancer mortality. In addition to traditional risk factors, measures of overall fitness including sarcopenia and body mass index (BMI) have been associated with clinical outcomes in lung cancer patients [2]. Sarcopenia, partly defined as a loss of skeletal muscle mass, occurs as a natural part of the aging process [3]. This process is mainly determined by two factors: the initial amount of muscle mass and the rate of loss over time [4]. Medical conditions such as cancer can contribute to and accelerate muscle loss leading to sarcopenia. In cancer patients, sarcopenia has been shown to lead to decreased overall survival (OS) and higher levels of morbidity [5]. 

Additional metrics of fitness including sarcopenia and BMI have also been used as prognostic factors in evaluating clinical outcomes across several disease processes. Specifically, in lung cancer, sarcopenia has been associated with worse postoperative outcomes and poorer survival [6]. Many of the previous studies assessing the relationship between sarcopenia and clinical outcomes have studied cancer treatments consisting of chemotherapy and surgical procedures [5,7]. Unlike sarcopenia, in prior literature BMI appears to be associated with improvements in outcomes in lung cancer patients treated similarly [8]. Currently, a paucity of data exists detailing the role of sarcopenia and other metrics of fitness in early stage lung cancer patients treated non-invasively with stereotactic body radiotherapy (SBRT).

SBRT is a cancer treatment that uses highly conformal radiation techniques to maximize the therapeutic ratio of delivering dose to tumor cells while minimizing the dose delivered to organs at risk (OARs) [9,10]. Existing literature indicates that there are minimal long-term toxicities associated with SBRT, excellent local control of primary tumors, and association with improvements in patient quality of life over other more invasive treatment modalities [11,12]. SBRT has become the standard of care treatment for medically inoperable patients with early-stage non-small cell lung cancer (NSCLC) and is a commonly used modality in the setting of multidisciplinary oncologic care [13,14].

Given the lack of data regarding the role of sarcopenia and other metrics of fitness in patients with NSCLC treated non-invasively with SBRT, we aimed to evaluate the potential role of sarcopenia, using normalized total psoas area (NTPA) as a surrogate prognostic factor in patients with NSCLC undergoing SBRT for definitive oncologic management.

## Materials and methods

This study was approved by the Institutional Review Board at our institution. We conducted a retrospective review of all patients who met eligibility criteria at our institution with early-stage, node-negative NSCLC treated with SBRT between 2013 and 2019. Eligibility criteria for study inclusion are as follows: (1) patients had biopsy-proven NSCLC, or, if medically unable to have a biopsy, were treated as NSCLC after multidisciplinary discussion; (2) the patient was treated with SBRT and met all requirements to be treated with SBRT per institutional protocol; (3) the patient had evaluable pretreatment imaging with either diagnostic CT, positron emission tomography (PET)/CT, or the CT obtained during radiation simulation; and (4) the patient had evaluable imaging obtained ≤1 month before treatment with SBRT. The analysis considered all consecutive patients at our institution.

Clinical variables of interest were determined a priori based upon author consensus and previously published literature. The following variables were defined and abstracted from the electronic medical record for analysis: age, sex, BMI, history of diabetes, hypogonadism, hyperthyroidism, osteoporosis, chronic kidney disease (CKD), HIV, and rheumatoid arthritis. The previously listed factors were abstracted and utilized to calculate a Charlson comorbidity index (CCI) score for each patient. Additionally, the following outcome variables were obtained: local failure, distant failure, vital status, and date of last follow-up or death.

Defining sarcopenia

**Figure 1:**
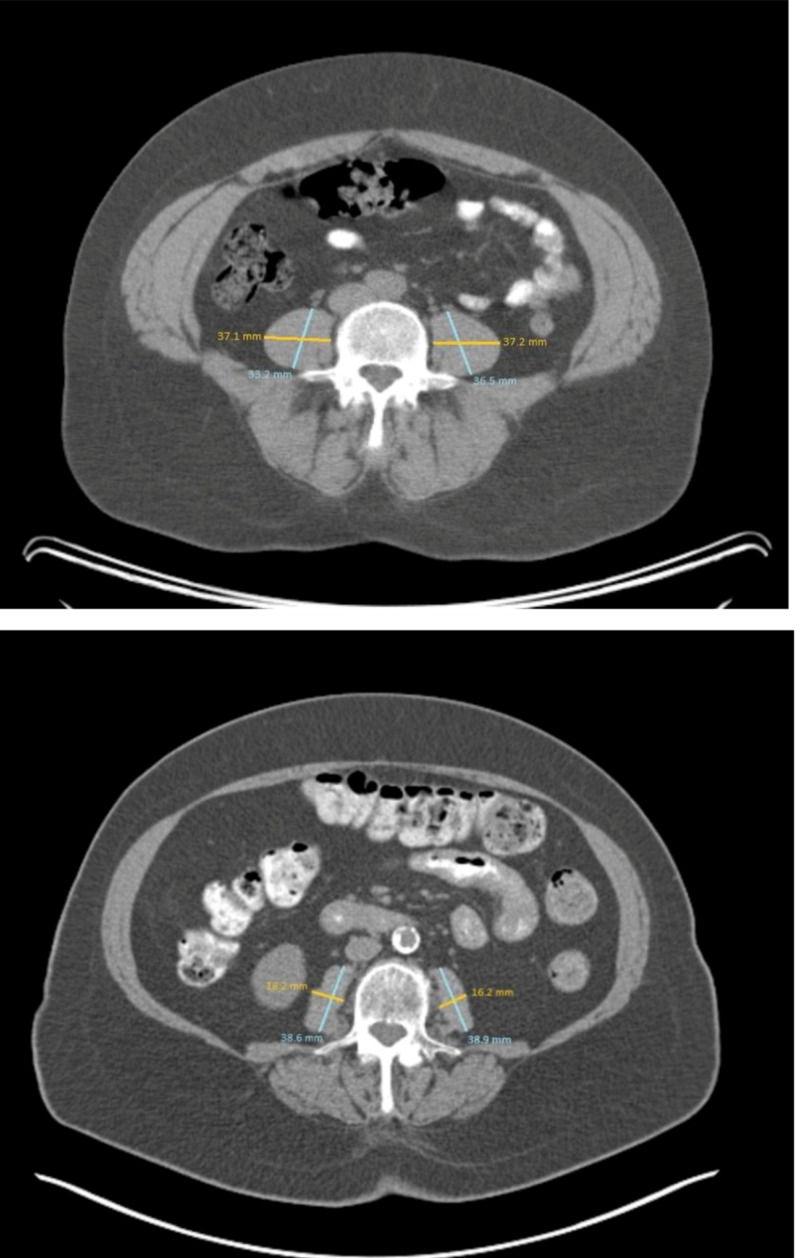
Representative measurement of the psoas muscle in a patient without and with sarcopenia

**Figure 2:**
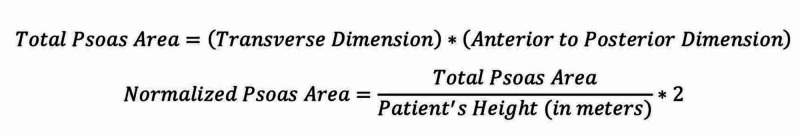
Formula to calculate normalized total psoas area

Sarcopenia was defined using NTPA for all patients included in the current study. Evaluable imaging studies including either a diagnostic CT, PET/CT, or radiation treatment planning CT obtained one month or sooner prior to SBRT were loaded into either PACS or Monaco Sim (Elekta, Stockholm, Sweden) and used for evaluation. To ensure uniformity, all measurements were taken at the level of the L3 vertebral body in the middle of the vertebral body. Once the psoas was identified at the level of L3, measurements were taken of the largest anterior-posterior and transverse dimension in both muscles (Figure 1). These values were multiplied to calculate the cross-sectional area at L3 expressed in mm^2^ (Figure 2). The larger value for each of the two psoas muscles measurements was chosen and normalized. Normalization was performed based on the unilateral psoas area, followed by division of the patient’s height in meters then multiplied by 2 to account for total psoas area [15]. The threshold for sarcopenia was dependent on sex, with NTPA <545 mm^2^/m^2^ for males and <385 mm^2^/m^2^ for females [16,17].

Statistical analysis

Baseline characteristics and descriptive statistics were summarized for the data set. Survival functions including OS, local failure free survival (LFS), progression free survival (PFS), and distant failure free survival (DFS) were evaluated using the Kaplan-Meier method. OS was defined as time from treatment with SBRT until death. PFS and LFS were defined as time from treatment with SBRT until first local recurrence (if any) or locoregional recurrence, respectively, while DFS was defined as time from treatment with SBRT until the development of distant metastatic disease. Log-rank tests and Cox-proportional hazards models (for categorical and continuous variables, respectively) were performed for Kaplan-Meier curve comparisons between stratifications for significant associations with survival. Covariates included in the model are as follows: presence of sarcopenia (as previously defined using NTPA and gender-specific values) age, CCI, sex, Karnofsky Performance Status score (KPS), and BMI. With regards to BMI, for analysis, we modeled this variable both as a continuous variable and a categorical variable. For the categorical comparison, we dichotomized the continuous variable into two groups based upon standardly recognized categories of BMI. We used the cut-off value of 18.5, with patients with BMI ≥18.5 considered normal weight, and patients with BMI <18.5 considered underweight. Statistical significance was set as p < 0.05. All analyses were performed on R version 3.5.2.

## Results

A total of 91 consecutive patients (out of 115) met criteria and were included in the analysis. Baseline characteristics and descriptive statistics were summarized and can be found in Table 1. Patients had a median age of 76 years and median KPS score of 80 (range: 60-100). Approximately 55% of patients were female and 79% of patients had T1 tumors (Table 1).

**Table 1:** Patient characteristics BMI, body mass index; KPS, Karnofsky Performance Status score

Characteristics	Total Patients (n = 91)
Age	
Median (year)	76
Range (year)	45-95
Gender	
Male (n, %)	41 (45%)
Female (n, %)	50 (55%)
Dose prescription	
Median	6,000 cGy
Range	4,500-6,000 cGy
Prescribed fractionation	
Median	5 fractions
Range	3-5 fractions
KPS	
Median	80
Range	60-100
Normalized total psoas area	
Median	531.6 mm^2^/m^2^
Range	90.4-1356.2 mm^2^/m^2^
Sarcopenia	
Yes (n, %)	39 (42.8%)
No	52 (57.2%)
BMI	
≥18.5 (n, %)	82 (90.2%)
<18.5 (n, %)	9 ( 9.8%)

The median radiation dose was 60 Gy (range: 45-60) and most commonly delivered in 5 fractions (range: 3-5). Median NTPA was 531.16 mm^2^/m^2^ (range: 90.4-1356.2). Based on sex and height adjusted values, 39 patients (42.8%) had sarcopenia. A total of nine (9.9%) patients were considered underweight (BMI < 18.5). A total of 26 patients experienced disease recurrence with 21 patients experiencing local failure only, 6 patients experiencing both local and distant failure, and 5 patients experiencing distant failure only.

**Figure 3:**
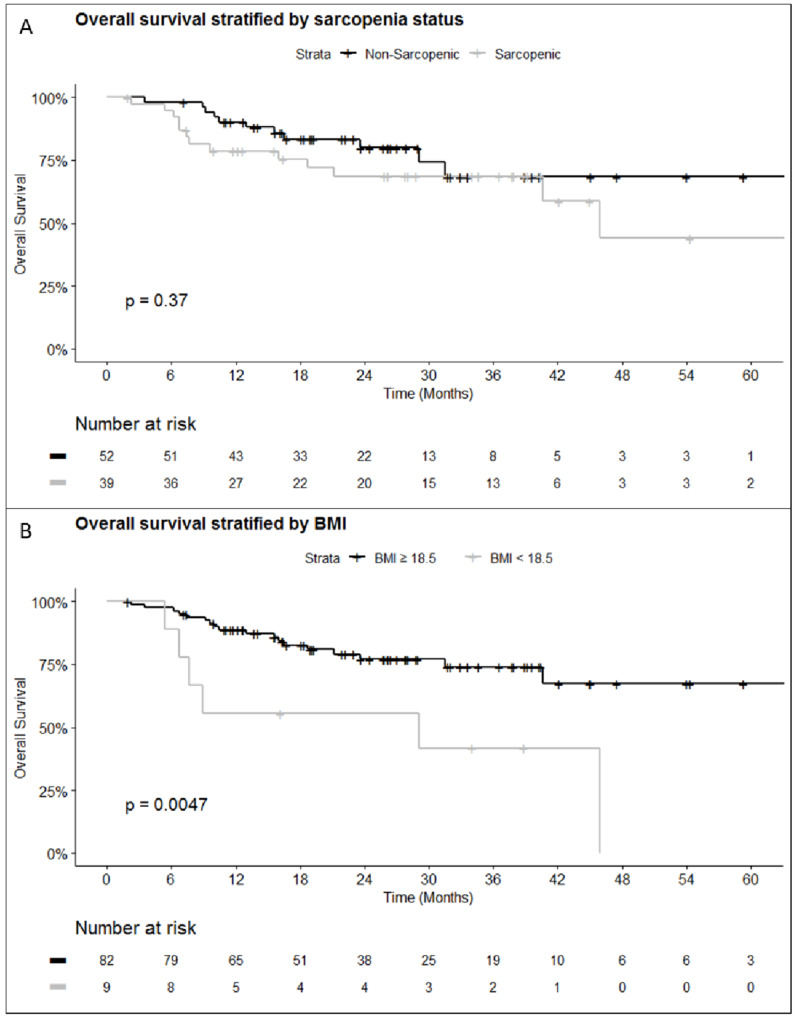
Overall survival by Kaplan-Meier (A) Overall survival stratified by sarcopenia status determined by sex-specific normalized total psoas area (male <545 mm^2^/m^2^; female <385 mm^2^/m^2^). (B) Overall survival stratified by body mass index (BMI) ≥18 or <18.

In the entire cohort, on multivariable analysis, higher CCI scores were associated with worse LFS (HR 1.2, 95% CI 1.1-1.4), DFS (HR 1.3, 95% CI 1.1-1.4), and PFS (HR 1.2, 95% CI 1.1-1.5) (Table 2). Despite this association, higher CCI scores were not found to be associated with worse OS (HR 1.1, 95% CI 0.96-1.2). Increasing BMI as a continuous variable was associated with better OS (HR 0.90, 95% CI 0.83-0.98), but had no association with either improvements or reductions in LFS, PFS, or DFS (Figure 3). When BMI was dichotomized and patients were grouped as underweight vs normal weight, OS was worse in patients with a BMI <18.5 (HR 3.5, 95% CI 1.3-8.9).

**Table 2:** Multivariate analysis of clinical variables and associations with oncologic outcomes BMI, body mass index; KPS, Karnofsky Performance Status score; CCI, Charlson comorbidity index

	Overall Survival	Local Failure Free Survival	Distant Failure Free Survival	Progression Free Survival
	HR (95% CI), p-value	HR (95% CI), p-value	HR (95% CI), p-value	HR (95% CI), p-value
Sarcopenia, yes vs no	1.3 (0.28-5.5) p = 0.75	0.61 (0.22-1.7), p = 0.35	1.2 (0.3-4.6), p= 0.81	0.66 (0.26-1.66), p = 0.37
BMI	0.90 (0.83-0.98), p = 0.006	0.99 (0.91-1.1), p = 0.46	0.91 (0.78-1.1), p = 0.24	0.96 (0.88-1.1), p = 0.36
KPS	0.97 (0.93-1.01), p = 0.26	0.98 (0.94-1.04), p = 0.72	0.97 (0.91-1.03), p = 0.37	0.98 (0.94-1.02), p = 0.42
CCI	(0.9-1.3), p = 0.28	1.2 (1.1-1.4), p = 0.024	1.3 (1.1-1.4), p = 0.008	1.2 (1.1-1.5), p = 0.006
Age	0.99 (0.94-1.03), p = 0.69	0.99 (0.95-1.04), p = 0.68	0.91 (0.85-0.98), p = 0.02	0.97 (0.93-1.01), p = 0.23

 

Sarcopenia (using NTPA and gender-specific values) was not associated with OS (HR 1.3, 95% CI 0.28-5.5), LFS (HR 0.61, 95% CI 0.22-1.7), or DFS (HR 1.2, 95% CI 0.3-4.6) or PFS (Figure 3). With a median follow-up of 23.4 months, median OS was 60, 60, and 45.9 months (p = 0.37) in all patients, non-sarcopenic patients, and sarcopenic patients, respectively. 

## Discussion

Our study is one of the first to assess the relationship between metrics of fitness and outcomes in a diverse population of patients with early-stage NSCLC treated non-invasively with SBRT. As hypothesized, we found no association between sarcopenia and OS or oncologic-specific outcomes in patients treated non-invasively with SBRT. In accordance with prior data regarding locally advanced NSCLC, we demonstrated a protective benefit from increasing BMI. Finally, we found no association between higher CCI scores and OS although there did appear to be an association with oncologic specific outcomes.

Sarcopenia has been shown to be associated with increased frailty and decreased quality of life [2]. It is a natural process of aging, however, and tends to be more prevalent in sicker patients with multiple comorbidities, especially in cancer patients. A study of NSCLC patients found close to 50% of patients had sarcopenia, indicating that a significant number of NSCLC patients are sarcopenic prior to undergoing cancer treatment. Sarcopenia has been shown to be a negative prognostic factor and is associated with increased morbidity and mortality in lung cancer patients undergoing therapy [5-7,18]. Previous studies have assessed the prognostic implications of sarcopenia in patients with lung cancer receiving multiple treatment modalities including chemotherapy and surgical procedures, but few studies have looked at SBRT. Therefore, there is a paucity of data on the value of sarcopenia as a prognostic factor in patients undergoing SBRT for NSCLC. 

The pathophysiology and risk factors for sarcopenia are complex and involve a multifactorial process including neurodegenerative processes, dysregulation of cytokine metabolism, changes and upregulation of the inflammatory cascade, reduction in anabolic hormone synthesis, and pre-existing fitness level [19]. Many of these factors are directly related to age. As an example, aging is associated with reductions in production and sensitivity to hormones including growth hormone and insulin like growth factor I as well as the production of the sex hormones androgens and estrogens. These factors can contribute to depletion of muscle mass and increase visceral fat all leading to sarcopenia. Cancer patients are at risk for multiple aspects of the sarcopenic cascade. Cancer patients tend to be older, have dysregulation of the inflammatory cascade, and are often de-conditioned secondary to the metabolic demands of the oncologic disease process. These are just several of the factors that contribute to sarcopenia in cancer patients, and this process may be accelerated by invasive systemic treatment modalities such as surgery and chemotherapy.

Several definitions and metrics have been developed and utilized to clinically define sarcopenia for research studies and patient application [20,21]. A consensus statement by the European Working Group on Sarcopenia in Older People (EWGSOP) recommends quantifying both skeletal muscle mass and function [3]. However, given the retrospective nature of the study, we have chosen to quantify skeletal muscle mass as a surrogate for sarcopenia as obtaining information on function was not feasible. Previously used methods to assess muscle mass include: bioelectrical impedance analysis (BIA), dual-energy X-ray absorptiometry (DEXA), four skinfold method, CT of L3, total psoas area, and NTPA [21,22]. As previously described, we choose to utilize NTPA as a surrogate for sarcopenia in our analysis. After consideration, the authors chose this method as every patient treated with SBRT requires pretreatment imaging and a dedicated simulation CT scan, which were evaluable for all patients. Additionally, given the retrospective nature of the study, total psoas area was deemed a reliable metric that could be applied post hoc to previously obtained imaging studies. 

The majority of the previous studies looking at the prognostic value of sarcopenia in lung cancer patients evaluated its use in patients treated definitively with surgical resection and chemotherapy. Suzuki et al. studied patients with stage I NSCLC who underwent complete surgical resection and the relationship of sarcopenia with clinical outcomes. They found that patients with sarcopenia had significantly worse outcomes than patients without sarcopenia, with differences in five-year survival of 72.8% versus 85.8% (p = 0.028) [7]. Cortellini et al. looked at the correlation between skeletal muscle mass and clinical outcomes in NSCLC patients treated with first-line chemotherapy [23]. On multivariate analysis, they found that baseline low skeletal muscle mass was a significant predictor of shorter PFS. They hypothesized that good nutritional status assists chemotherapy delivery, without the need for discontinuation, resulting in better effectiveness [23]. Lastly, in a study by Nakamura et al. published in the Journal of Clinical Oncology, the authors reported that sarcopenia was an independent unfavorable prognostic factor in patients undergoing curative resection for NSCLC [24].

In contrast to the current analysis, these previous studies did not look specifically at SBRT as a primary treatment modality. Additionally, the results from our analysis differ from those previously discussed as we did not demonstrate similar associations between sarcopenia and clinical outcomes. Compared with the data by Suzuki et al. we did not show a similar association between OS and sarcopenia. This could be explained by several factors: (1) we defined sarcopenia differently than the authors, (2) surgical resection is known to have higher morbidity than SBRT and thus the impact of pre-existing sarcopenia and deconditioning has a greater impact on OS, or (3) our patient populations are inherently different. We believe this difference of effect is primarily due to the non-invasive nature of SBRT. Unlike systemic chemotherapy and surgical interventions, SBRT does not create the same systemic toxic effects and thus may require less physiological reserve. As such, even in patients who are sarcopenic, SBRT may not increase mortality or diminish oncologic outcomes. Furthermore, based on our results, we hypothesize that baseline fitness is of less importance when being treated non-invasively with SBRT compared with either surgery, chemotherapy, or multimodality therapies.

Despite this, our findings differ from that of a recent study by Matsuo et al. that also looked at the association between sarcopenia and 186 patients with early-stage, node-negative NSCLC treated definitively with SBRT. The authors demonstrated that although low skeletal muscle mass is not associated with increased lung cancer death or treatment related toxicity, it is a significant risk factor for non-lung cancer mortality [25]. Therefore, Matsuo et al. implied that pre-existing fitness and overall health do impact outcomes, even in patients treated non-invasively with SBRT. The differences seen between their study and our own may be due to several differences in the patient populations that were studied. Another potential factor that may explain the differences in our results compared to those found by Matsuo and colleagues may be attributable to study size and follow-up time. Our study included 91 total patients, with only 39 being classified as sarcopenic compared to a total of 186 patients with 94 being qualified as having low skeletal muscle mass in the study by Matsua. Additionally, we report clinical outcomes with a median follow-up of 23.4 months compared with 4.6 years. The additional time for follow-up may have allowed enough time for clinically and statistically meaningful differences to be observed between the study populations. Another factor that may contribute to this difference is that we choose to analyze OS by presence or absence of sarcopenia and did not specifically look at non-lung cancer mortality. Lastly, the study by Matsuo et al. most likely represents a more homogeneous patient population than the population treated in our clinic and may account for the differences in outcomes between the two studies.

With regards to additional metrics of fitness, including the BMI, we dichotomized the continuous variable into two groups based upon the standard BMI categories. We used the cut-off value of 18.5, with patients with BMI ≥18.5 considered normal weight and patients with BMI <18.5 considered underweight. When these criteria were applied to our cohort, nine patients were considered underweight. These patients had statistically worse OS compared with patients with BMI ≥18.5. We recognize that this is a small percentage of the total cohort, but this value reflects the general rate of underweight patients in our clinic. Additionally, when modeled continuously, increasing BMI was associated with improved OS, with an HR of 0.90 (95% CI 0.83-0.98, p = 0.006).

Our data indicate that lower BMI was associated with poorer OS and increasing BMI was protective with improved OS. Of all variables abstracted from the electronic medical record (EMR) for data analysis, this was the only variable associated with OS in any direction. It is unclear if higher BMI is somehow protective in this cohort, or if BMI is a better clinical predictor of outcomes compared with sarcopenia, as measured using NTPA. The role of BMI in patients with lung cancer is complicated, as previous studies have shown a protective benefit in patients with BMI ≥30. Prior surgical series have shown decreased operative mortality and respiratory complications among overweight/obese patients with NSCLC undergoing lobectomy [8]. More recently, Kichenadasse et al. report that increasing BMI is associated with improved OS in patients with advanced NSCLC treated with immune checkpoint inhibition [26]. Although this represents a very different patient population than our own, it again provides evidence that increasing BMI may be protective in patients with lung cancer at any stage. As with sarcopenia, limited data exist assessing the association between BMI and outcomes in patients with NSCLC treated with SBRT. Perhaps, in accordance with prior studies, higher BMI remains protective even in patients treated non-invasively with SBRT [27]. When taken together, our data and prior data suggest a protective benefit to increasing BMI in lung cancer patients at all stages treated with multiple modalities including SBRT, chemoradiotherapy, surgical resection, and immune checkpoint inhibition.

A final category of patients should also be discussed that are both sarcopenic and obese, a conditioned termed sarcopenic obesity [28]. A recent review of published literature on the clinical implications of sarcopenic obesity in cancer patients identified 14 studies addressing this patient population. While the authors conclude there is a high degree of heterogeneity in how sarcopenic obesity is defined, there is evidence to suggest that cancer patients with sarcopenic obesity have shorter survivals [29]. While we did not specifically address this group of patients within our analysis given the protective benefit of higher BMI demonstrated in our study and in prior literature, it is unclear how patients with NSCLC who have sarcopenic obesity would compare to patients treated similarly with normal fitness metrics. Future prospective work in larger patient populations is needed to further define the clinical implications of sarcopenic obese patients undergoing SBRT for the treatment of NSCLC.

Lastly, as predicted, higher CCI scores were associated with worse outcomes, including PFS, LFS, and DFS. Interestingly, and in contrast to the authors’ a priori hypothesis, CCI was not associated with worse OS. One likely explanation again is predicated on the non-invasive treatment approach with SBRT. In contrast to more invasive approaches such as lobectomy, pre-existing medical comorbidity (as assessed by CCI) and deconditioning (reflected by sarcopenia status) may have no or limited impact on outcomes in patients treated with SBRT. Also, given that SBRT is often reserved for medically inoperable patients with multiple medical comorbidities any effect of CCI may be lost as the majority of patients included in our study had high CCI scores (median CCI of 7). Again, as with BMI, further confirmation of this finding should be reproduced in future research to help elucidate potential mechanisms and interactions between CCI, BMI, and sarcopenia in this patient population. 

The current study has several strengths. All patients had evaluable imaging within one month of beginning SBRT, making a uniform time point for psoas measurement and calculation of NTPA. Additionally, all measurements were taken by a single of the study’s authors ensuring precision and repeatability of measurements. Lastly, the study includes 91 patients treated between 2013 and 2019 with modern SBRT techniques (volumetric radiation prescription and daily image guidance), which provides an accurate representation of the potential morbidity associated with SBRT and is applicable to current practice. Limitations include the retrospective nature of the study and sample size, which may not accurately provide data to characterize the impact of sarcopenia on patients treated with SBRT. The analysis is also limited by the short median follow-up time of 23.4 months, which may not have allowed time for enough events to occur in a disease with a median survival of approximately four to five years. Additionally, we do not present detailed information on second-line treatments used for disease recurrence or treatment failure. This could affect survival outcomes and thus influence our results depending on which second-line treatments were used. Even though our series represents consecutive patients treated at our institution, many are referred for treatment and return to care in the community. Given this, we are limited in our ability to present detailed information on second-line treatments. Finally, given the retrospective nature of the study, only muscle mass not muscle function was quantified as has been suggested by the EWGSOP and in prior series.

## Conclusions

Our study adds to the limited data that exist characterizing the utility of sarcopenia as a prognostic factor for lung cancer patients undergoing non-invasive treatment with SBRT and questions the use of low skeletal muscle mass as a prognostic factor in this patient population. We demonstrate no association between sarcopenia, as defined using NTPA, and clinical outcomes. As hypothesized, higher CCI scores were associated with worse clinical outcomes. However, based on our data, increasing BMI appears to be a predictor of outcomes. Perhaps BMI would serve as a better clinical predictor compared with sarcopenia in patients treated with SBRT. Future prospective work is needed to further define the role of sarcopenia, BMI, and other metrics of overall fitness in patients with NSCLC treated non-invasively with SBRT.
